# Cytopathological quantification of NORs using artificial intelligence to oral cancer screening

**DOI:** 10.1590/1807-3107bor-2025.vol39.056

**Published:** 2025-05-12

**Authors:** Tatiana Wannmacher LEPPER, Luara Nascimento do AMARAL, Ana Laura Ferrares ESPINOSA, Igor Cavalcante GUEDES, Maikel Maciel RÖNNAU, Natália Batista DAROIT, Alex Nogueira HAAS, Fernanda VISIOLI, Manuel Menezes de OLIVEIRA, Pantelis Varvaki RADOS

**Affiliations:** (a) Universidade Federal do Rio Grande do Sul - UFRGS, School of Dentistry, Department of Oral Pathology, Porto Alegre, RS, Brazil.; (b) Universidade Federal do Rio Grande do Sul - UFRGS, Informatics Institute, Porto Alegre, RS, Brazil.; (c) Universidade Federal do Rio Grande do Sul - UFRGS, School of Dentistry, Department of Periodontology, Porto Alegre, RS, Brasil.

**Keywords:** Mouth Neoplasms, Early Detection of Cancer, Cytology, Artificial Intelligence

## Abstract

Oral squamous cell carcinoma (OSCC) remains the most prevalent neoplasm of the head and neck. In recent decades, the incidence and prevalence of OSCC have not significantly changed, highlighting the critical need to develop and implement new risk assessment measures. The present study aimed to define argyrophilic proteins of the nucleolar organizer region (AgNOR) cut-off risk points by oral exfoliative cytological smears comparing specialized humans with a convolutional neural network (CNN) system AgNOR Slide-Image Examiner. This study included four experimental groups: control, exposure to carcinogens (alcohol and tobacco), oral potentially malignant disorders, and OSCC. In the first phase, 50 cells were used for AgNOR quantification. In the second phase, AgNOR quantification was established in an automated manner using an AgNOR System – Slide Examiner (captured – bounding-boxed – CNN analysis). In phase 1, the cut-off point for considering a smear as suspicious was established at 3.69 AgNORs/nucleus with sensitivity of 86%, specificity of 93%, and accuracy of 90%. In phase 2, the analysis of the intraclass correlation coefficient of AgNORs attributed to the system and human was 0.896 (95% confidence interval = 0.875–0.915; p < 0.0001), and this quantification with the CNN was 20 min compared to 67 h, considering human analysis. The AgNOR Slide-Image Examiner successfully differentiated the nuclei and accurately quantified the number of NORs in oral cytological smears. The cut-off risk point of 3.69 AgNOR/nucleus indicates a suspicious sample may contribute to improvements in oral cancer screening.

## Introduction

Oral squamous cell carcinoma (OSCC) is the most common malignant neoplasm of the oral cavity,^1^ developing in the epithelial lining of the oral mucosa. According to the World Health Organization,^2^ OSCC is the most prevalent neoplasm of the head and neck. The incidence and mortality rates of OSCC vary within each country.^3^ Owing to the late diagnosis, approximately 80% of OSCC cases are detected at advanced stages, which significantly reduces survival rates.^4^

OSCC can be preceded by oral potentially malignant disorders (OPMD), with the most common clinical presentation being leukoplakia, with an estimated prevalence of 1.7% to 2.7.^5^ The transformation rate can vary from 1.4 to 49%.^6^ Although leukoplakia is a significant clinical precursor, the percentage of malignant transformations remains highly unpredictable. Furthermore, many cases of OSCC may not originate from these lesions in patients exposed to classical oral risk factors such as alcohol and smoking.^7^ These potentially malignant disorders are often biopsied, and in most cases, multiple follow-up biopsies are indicated.^8^

Visual clinical examination followed by biopsy and histopathological examination are the gold standards for diagnosing OPMD and OSCC.^9^ However, choosing solely through clinical criteria in which the lesion area of OPMD should be biopsied remains a challenge in clinical practice,^10^as well as the difficulty in making a clinical decision regarding new interventions during the clinical follow-up of OPMD.^11^ In recent decades, this context has not undergone major changes, highlighting the importance of measures for this pathology.^9,11^

Minimally invasive methods, such as cytopathology associated with physical examination, have been studied to develop protocols that help monitor individuals at risk of developing oral cancer and their association with computer-assisted systems.^12,13^

An increase in the cell proliferation rate reduces the possibility of DNA repair, contributing to multiple genetic events in carcinogenesis.^14^ Quantification of the argyrophilic proteins of the nucleolar organizer regions (AgNORs), non-histone proteins associated with ribosomal genes, is a marker of cell proliferation that can be measured in cytopathologic and histopathologic samples and is increased in individuals who consume alcohol and tobacco.^15,16^ Previous studies evaluating AgNORs aimed to diagnose oral lesions and observed a variation in the mean AgNORs/nucleus of normal mucosa to squamous cell carcinomas from 2.81 to 8.69.^17-23^

Some studies establish an AgNOR/nucleus cut-off point for distinguishing between benign and malignant lesions, with an average value ranging from 3.38 to 6.52.^15,21,23^ Therefore, current studies have demonstrated that cell capture systems integrated with diagnostic imaging algorithms can distinguish between high- and low-risk oral lesions more precisely and accelerate the process.^13,14,24,25^

This study aimed to define the mean AgNOR per nucleus and establish cut-off malignancy risk values in individuals exposed to alcohol and tobacco compared with individuals diagnosed with OPMD and OSCC. In addition, AgNOR manual specialized human counting and automated counting were compared.

## Methods

### Study design and participants

**Figure 1:**
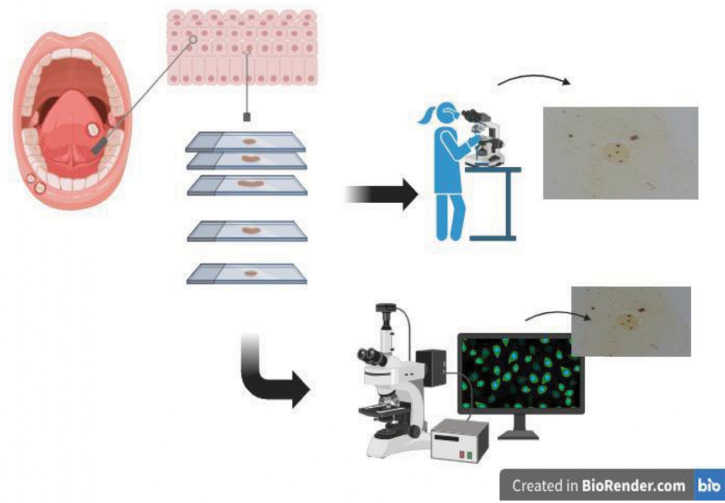
Scheme of cytopathological collection of oral mucosal smears and quantitative assessment of AgNOR in phase 1 by human manual quantification and in phase 2 by CNN quantification.

This cross-sectional study was conducted in two phases. In phase 1, manual human analysis of the pattern of cell proliferation activity was performed on cells obtained from the oral mucosa through exfoliative cytology subjected to the AgNOR technique. In phase 2, AgNOR quantification was performed automatically using the AgNOR System – Slide Examiner, a convolutional neural network (CNN)-based approach for joint segmentation and quantification defined, trained, and tested by the same research group and detailed in a previous study,^13^ as shown in Figure 1.

This study was approved by the Research Committee of the Faculty of Dentistry and the Research Ethics Committee of the Federal University of Rio Grande do Sul, Brazil (study number CAE 39212420.9.0000.5347). This study was conducted in accordance with the principles of the Declaration of Helsinki. The study was performed from 2017 to 2022 at the Department of Oral Medicine and Oral Pathology and included 123 patients in two phases. All patients were invited to participate in the study. The researchers explained the risks and benefits, addressed questions, and presented the consent form. Clinical and cytopathological procedures were performed after informed consent was obtained.

Patients of both sexes aged > 18 years were included in the study. Patients with visible lesions in the oral mucosa (except for periodontal disease), previous or current history of malignant or benign tumors, radiotherapy and/or chemotherapy, and use of fixed orthodontic appliances were excluded.

Our research focused on the cytopathological changes that indicate the potential for malignancy in oral tissues. Control groups (CG) were used as references: the non-exposed/non-lesional group was used as a negative control, and the OSCC group (OSCCG) was used as a positive control. The exposed group (EG) and OPMD group (OPMDG) were included to detect early microscopic alterations preceding clinical manifestations, with the aim of secondary prevention through population screening. Patients were enrolled in the following sample groups:

CG: individuals who did not have clinically detectable lesions, never smoked, and did not consume up to 30 g of alcohol per day

EG: individuals who did not have a clinically detectable lesion, smoked, or consumed alcoholic beverages

OPMDG: individuals with clinically detectable lesions, such as leukoplakia, erythroplakia, or leukoerythroplakia, confirmed by histopathological examination of epithelial dysplasia or non-dysplastic lesions

OSCCG: individuals with clinically suspected oral cancer, confirmed by biopsy and histopathology

### Clinical procedures

The participants were interviewed using a questionnaire to collect their sociodemographic data. All participants underwent clinical examination, sitting in the proper position with artificial light. A gauze was used to dry the mucosa, and an oral mirror was used to improve visualization. Dentures and removable appliances were removed during the inspection. The physical examination sequence was as follows: lips, buccal mucosa, alveolar ridges, hard and soft palates, tongue, and floor of the mouth. Patients who presented with lesions requiring biopsy were subsequently subjected to the procedure under local anesthesia. The obtained material was fixed in 10% buffered formalin, processed routinely for microscopic examination of inclusions in paraffin, and stained with hematoxylin and eosin. The diagnosis was based on the criteria established by the World Health Organization.^26^

### Cytopathological collection

Four trained researchers performed the collection. Cells were obtained using a sterile cytobrush^®^ with 10 rotational movements over the area of choice. In the CG and EG, samples were collected from the right lateral border of the tongue; in OPMDG and OSCCG, the collection was performed over the lesion, except in ulcerated lesions, in which cells from the periphery of the lesion were collected to avoid bleeding or collection of necrotic material. Cytopathological analysis was performed before biopsy in the lesion groups. The material was spread by rotational movement over three glass slides, fixed in absolute alcohol, and stored at 4ºC until AgNOR staining was performed. All cytological analyses were performed using a binocular microscope (Olympus Optical Co., Tokyo, Japan, model CX41RF), and the images were captured with Image J software on a trinocular microscope (Nikon Eclipse Si, CFI60 infinity optical system 0.55X relay lens), and the images were captured with Capture value 2.3 i5.

### Sample size calculation

The sample size was calculated based on Paiva et al.^23^ using Winpepi^®^, considering a mean expected inter-group difference of 0.59 AgNORs/nucleus between the CG (standard deviation [SD] 0.56) and OSCCG (SD 0.58), significance level of 5%, and power of 80%, resulting in 15 participants per group being required. Considering a drop-out rate of 10%, 17 participants were recruited per group.

### Calibration

Cytopathological and histopathological microscopic evaluations were performed by three independent examiners (TWL, LNA, and PVR). Inter-examiner calibration was performed through 10 repeated evaluations of 25 smears with an 8-day interval between them. The inter-examiner reliability was > 80%.

### Cytopathological analysis parameters

*Phase 1*: Quantitative Assessment of Human and Manual AgNOR

Cytological smears were subjected to the AgNOR technique.^27^ The NORs were identified as well-defined black dots in the nucleus, with any overlapping or fused dots considered a single structure quantified in the first 50 cells after 1,000× oil immersion^.^ The analysis criteria were those previously described by Crocker et al.^28^ Overlapping cells, non-monolayered areas, and background areas were visually detected and automatically excluded from the analysis. The mean number of NORs/nucleus and the mean percentage of 1–13 NORs/nucleus were determined.

*Phase 2*: Quantitative Evaluation of AgNOR by CNN

**Figure 2:**
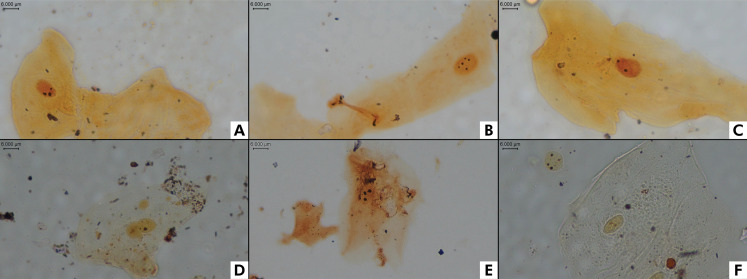
Cytosmear examples. Adequate samples for analysis: A) Cell containing 3 NORs/nucleus. B) Cell containing 5 NORs/nucleus. C) Cell containing 2 NORs/nucleus. Inadequate samples that were excluded from the study: D) Nuclear overlap and artifacts in the sample. E) Nucleus with poorly defined margin. F) Cell on the left: ill-defined cytoplasm. Cell on the right: nucleus without labeling for NORs. AgNOR. 1000×, oil immersion.

**Figure 3:**
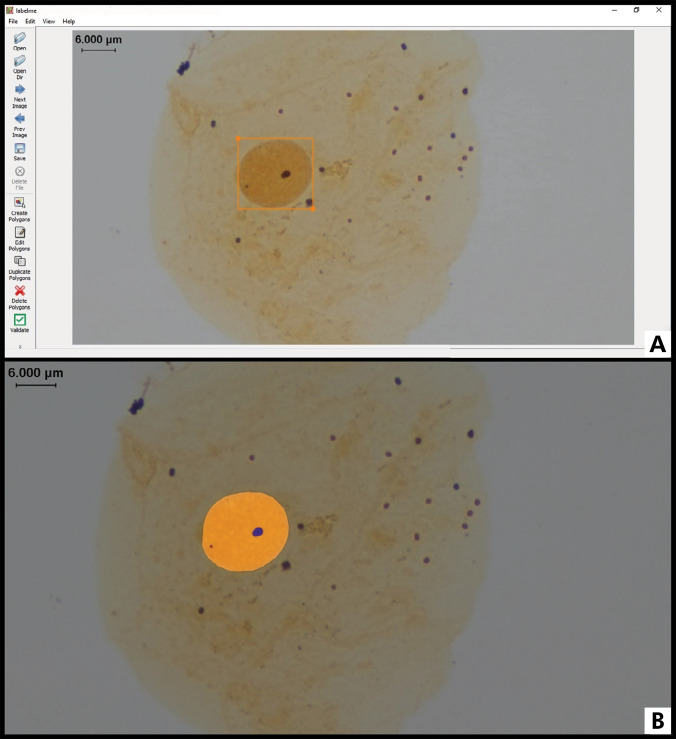
Quantification of NORs using the LabelMe system created by this group research (Ronnau et al.13) with segmentation using a bounding box. The bounding box technique was employed, digitally marking a rectangle with minimal dimensions to determine the nuclear area containing black dots corresponding to AgNORs. Demarcations were performed until reaching the first 50 cells well distended and not overlapping for posterior analysis by CNN. Cells bounding-boxed should present a clearly identified contour of the nuclear area. Nucleus overlapped or nuclear membrane presenting overlapping black dots were excluded. A: Bounding box around each selected nucleus. B: Identification of nuclei and NORs performed by the system.

Cytopathological smears were captured using Nikon Eclipse Si trinocular-specific software, a CFI60 infinity optical system, and a 0.55X lens with 400× magnification. A database of cell images was created for the four experimental groups, saved with the TIF or JPEG extension, following the same numerical registration per individual. Part of the obtained images was segmented using a bounding box to maintain the quantification of the first 50 cells for analysis by the CNN, LabelMe system, and AgNOR System – Slide Examiner (Figure 2). The same criteria described by Crocker et al.28 were used for human analysis. Overlapping cells and non-monolayered or background areas were visually detected, not segmented (bounding-boxed), and automatically excluded from the analysis (Figure 3). These images were then presented to the reading system, and through algorithms, the images were verified by searching for pre-established signals to determine the average number of NORs/nucleus and quantity from 1 to 4 and > 5 NORs/nucleus.

### Statistical analysis

All statistical analyses were conducted using the Stata software. The unit of analysis was the individual. The level of significance was set at 5%.

The agreement between human analysis and the AgNOR System – Slide Examiner was assessed using the intraclass correlation coefficient (ICC). An ICC above 0.75 indicated good reliability between the two quantification analyses.

The diagnostic accuracy of the human analysis was determined by fitting logistic regression models; thereafter, the sensitivity, specificity, and positive and negative predictive values were estimated. In addition, the area under the curve (AUC) was calculated to quantify the probability of AgNOR counts to distinguish different groups.

The optimal cut-off point was estimated using the cutpt command in Stata. This method estimates the optimal cut-off point for a diagnostic test by determining the cut-off point on the receiver operating characteristic curve closest to the point with perfect sensitivity and specificity. Specifically, in our case, this command determined the best cut-off for AgNOR counts to predict oral lesions.

## Results

### Phase 1

A total of 56 participants were included in this phase. There were 56.5% men and 43.5% women; the mean age was 57.5 years (SD ± 11.4 years). Of these, 15 were from the CG, 12 from the EG, 15 from the OPMDG, and 14 from the OSCCG.

The mean number of AgNORs was 2.89 ± 0.62 in the CG, 3.67 ± 1.03 in the EG, 3.25 ± 0.63 in the OPMDG, and 4.08 ± 0.46 in the OSCCG. Regarding AgNOR per nucleus, the CG presented a higher percentage of two NORs per nucleus, while the OSCCG and GE presented a higher percentage of more than five NORs per nucleus. The OPMDG showed the highest percentage, with three NORs/nucleus. There was a progressive increase in the percentage of nuclei with more NORs from CG to OPMDG and OSCCG and a decrease in the number of nuclei with one or two NORs from OSCCG to CG (Table 1).

**Table 1:** Mean AgNORs/nucleus and percentage of cells with 1 to 5 NORs per nucleus of all experimental groups in phase 1.

Reference groups	Mean ± SD	%1AgNOR	%2AgNOR	%3AgNOR	%4AgNOR	%5AgNOR
	AgNORs/nucleus					
CG	2.89 ± 0.62	13.3	29.6*	26.1	20.1	11.1
EG	3.67 ± 1.03	8.1	17	24.1	21.1	29.7*
OPMDG	3.25 ± 0.63	8.4	20	31.5*	23.6	16.5
OSCCG	4.08 ± 0.46	3.6	12.6	23.4	29	31.4*

CG: control group. EG: exposed group. OPMDG: oral potentially malignant disorder group. OSCCG: oral squamous cell carcinoma group. SD: standard deviation. AUC: area under curve. *higher frequency data.

Diagnostic accuracies are shown in Table 2. Regarding the mean AgNOR in all experimental groups, the best accuracy was observed in differentiating OSCCG from OPMDG and CG patients (AUC 0.85 and 0.93, respectively).

**Table 2:** Diagnostic accuracy of mean AgNOR of oral mucosa smears.

Comparison group	Sensitivity	Specificity	PPV	NPV	AUC
CG					
EG	66.7	73.3	66.7	73.3	0.75
OPMDG	53.3	66.7	61.5	58.2	0.65
OSCCG	92.7	80.0	81.2	92.3	0.93
EG					
OPMDG	73.3	50.0	64.7	60.0	0.65
OSCCG	85.7	50.0	66.7	75.0	0.61
OPMDG					
OSCCG	85.7	73.3	75.0	84.6	0.85

CG: control group. EG: exposed group. OPMDG: oral potentially malignant disorder group. OSCCG: oral squamous cell carcinoma group. PPV: positive predictive value. NPV: negative predictive value. AUC: area under curve.

The empirical cut-off point of AgNOR for predicting OSCCG (control patients as the reference category) was equal to 3.69 (Table 3), with an AUC of 0.90. A similar cut-off of 3.67 (AUC 0.68) and 3.68 (AUC 0.80) was estimated for OSCCG versus EG and OSCCG versus OPMDG, respectively.

**Table 3:** Empirical optimal cut-off points for mean AgNOR of oral mucosa smears.

Comparison group	Cut-off	Sensitivity at cut-off	Specificity at cut-off	AUC
CG				
EG	3.18	0.75	0.73	0.74
OPMDG	2.96	0.60	0,67	0.63
OSCCG	3.69	0.86	0.93	0.90
EG				
OPMDG	Not computed			
OSCCG	3.67	0.86	0.50	0.68
OPMDG				
OSCCG	3.68	0.86	0.73	0.80

CG: control group. EG: exposed group. OPMDG: oral potentially malignant disorder group. OSCC: oral squamous cell carcinoma group. AUC: area under curve.

### Phase 2

This phase included 67 individuals; a total of 3108 nuclei were bounding-boxed, and 2.845 AgNOR stains were counted, all with cytopathological material analyzed by humans and automated systems to identify AgNORs. Of these individuals, 26 were from the CG, 29 from the EG, 2 from the OPMDG, and 10 from the OSCCG. The mean number of AgNORs was 2.89 ± 0.62 in the CG, 3.67 ± 1.03 in the EG, 3.25 ± 0.63 in the OPMDG, and 4.08 ± 0.46 in the OSCCG.

During the human quantification of AgNORs, one smear was evaluated per hour, and 50 images/cells per patient were analyzed. The bounding-box step required approximately 20 min for each folder of 50 images. The “AgNOR Slide-Image Examiner” performed the AgNOR quantification of all images/folders in 10 min, with an Excel file output reporting the mean number of NORs per nucleus and the number of nuclei with 1, 2, 3, 4, and 5+ NORs per nucleus.

The results of the comparison of the total number of images evaluated by the human and CNN systems are presented in Table 4. The system discharged 214 bound-boxed nuclei. The percentage of nuclei missed by this system was 6.88%. The ICC analysis of the distribution of AgNORs per nucleus indicated good reliability between the AgNORs assigned by the system and those by humans (ICC = 0.896 [95% confidence interval = 0.875–0.915]), considering the bounding-boxed cells versus human quantification of the same cells.

**Table 4:** Percentage (%) of cells with 1 to 5 NORs per nucleus of all experimental groups in phase 2. General count of AgNORs of human and CNN AgNOR Slide-Image Examiner.

Reference group	Total count of AgNOR (H/CNN)	%1AgNOR (H/CNN)	%2AgNOR (H/CNN)	%3AgNOR (H/CNN)	%4AgNOR (H/CNN)	%5AgNOR (H/CNN)	ICC
CG	1246/722*	210/96	332/183	366/214	215/142	123/87	0.57
EG	882/617*	142/90	222/149	315/184	183/98	120/96	0.62
OPMDG	240/181**	20/16	44/36	54/40	66/50	56/39	0.75
OSCCG	379/299**	35/38	86/71	107/83	50/68	83/67	0.78

CG: control group. EG: exposed group. OPMDG: oral potentially malignant disorder group. OSCC: oral squamous cell carcinoma group. H/CNN: human/convolutional neural network automated analysis (AgNOR Image Examiner). * moderate reliability. ** good reliability. ICC: intraclass correlation coefficient.

## Discussion

In this cytopathological study using AgNOR staining through conventional human analysis, the cut-off point of 3.69 AgNORs/nucleus with an accuracy of 90% in OSCC samples was demonstrated. In the lesion groups, more than 30% of the nuclei contained more than three NORs. These values suggest cut-off points for the suspicion of malignancy in AgNOR-stained cytopathological smears from the oral cavity, confirming that cytopathology may be used as a screening tool.^11,29^

One of our main findings, supported by previous studies, is the increase in the mean AgNOR/nucleus in the OSCCG compared with the OPMDG and in the OPMDG compared with the CG. Remmerbach et al.^15^ reported the highest mean of OSCC AgNORs/nucleus (8.82 ± 2.60); however, the risk of malignancy cut-off point was lower, at 4.8 AgNORs/nucleus. Paiva et al.^23^ defined the cut-off point as 3.38, a result similar to our findings. Tumor malignancy grading and staging were not categorized in these studies, which may explain the observed numerical variations. Nevertheless, a high number of AgNORs per nucleus is a reliable marker for oral cancer screening. In this context, the biological plausibility is that an increased proliferation rate reduces the time for DNA repair mechanisms, causing DNA to exist in single strands, making it more vulnerable to damage and ultimately contributing to multiple and cumulative genetic events in carcinogenesis.^14^

Rajput and Tupkari^17^ suggested that the higher AgNOR/nucleus mean for the OSCC group (8.82) found by Remmerbach et al.^15^ may be due to a lower staining time (20 min) compared with their results (5.38 using 55 min). They argued that prolonged silver deposition may cause higher chemical element precipitation and the union of small-sized dot-like precipitations, ultimately accounting for lower amounts of NOR/nucleus. This argument also applies to the control group means when comparing the studies (2.28 vs. 2.56), which showed no considerable differences. Conversely, Jajodia et al.^21^ and Sowmya et al.^20^ used a 40-min silver staining time, resulting in high means of OSCC AgNORs/nucleus (7.7 and 7.67, respectively), similar to Remmerbach et al.,^15^ who used a 20-min sample staining time.

Establishing reference values for healthy tissues is crucial for effective screening of diseases. In the present study, the mean number of AgNORs per nucleus in the control group was 2.89 (± 0.62). Various cytopathological collection methods, such as wooden or metal spatulas, toothbrushes, and specific cytological brushes (Cytobrush^®^, Medibrush^®^), can be employed. Jajodia et al.,^21^ in the same group, reported a mean of 4.9 AgNORs per nucleus using a toothbrush for collection, which may have accessed the deeper, more proliferative layers of the epithelium, thereby increasing the marker count. The preparation could not interfere with the results, as evidenced by the study by Paiva et al.,^23^ which demonstrated a mean of 2.86 (±0.56) AgNORs per nucleus using liquid-based cytology (LBC), comparable to other studies employing conventional cytopathology (CC) with similar results (Table 5).

**Table 5:** Comparative analysis of studies using AgNOR and oral cytopathology.

Variables	Remmerbach, 2003^15^	Rajput, 2010^17^	Sharma, 2012^18^	Gonzalez Segura, 2015^19^	Sowmya, 2017^20^	Jajodia, 2017^21^	Silva, 2018^22^	Paiva, 2022^23^	Our study
Collection method	Cytobrush^®^	Cytobrush^®^	Cytobrush^®^	Medibrush^®^	Cytobrush^®^	Baby nylon toothbrush	Cytobrush^®^	Cytobrush^®^	Cytobrush^®^
						CC		LBC	
Agnor/Nucleus mean									
Control Group	2.28 (±1.7)	2.56 (±0.31)	2.91 (±0.23)	3.80 (±0.80)	3.51 (±1.62)	4.9	3.36 (± 0.89)	2.86 (± 0.56)	2.89 (± 0.62)
Subsite	Buccal mucosa	NI	Buccal mucosa, Floor of the mouth, Palate	Lesion opposite side	NI	NI	Tongue border	NI	Tongue border
							3.93 (± 0.14)		
							Floor of the mouth		
Exposed group (exposed substance)	-	-	3.35 (±0.38)	-	-	-	3.72 (± 0.94)	3.38 (± 0.71)	3.67 (± 1.03)
			Tobacco				Tongue border	Tobacco/ alcohol	Tobacco/ alcohol
			3.38 (±0.39)				4.31 (± 0.13)		
			Tobacco chewer				Floor of the mouth		
							Tobacco/ alcohol		
OPMDG (Leukoplakia)	3.79 (±0.62)	-	-	6.38 (±1.08)	5.66 (±2.51)	6.3	3.58 (± 0.13)	-	3.25 (± 0.63)
OSCCG	8.82 (±2.60)	5.38 (±0.34)	4.31 (±0.32)	6.49 (±1.21)	7.67 (±2.24)	7.7	4.16 (± 0.16)	3.45 (± 0.58)	4.08 (± 0.46)
Malignant suspect cut-off point	4.8	-	-	-	-	6.5	-	3.38	3.69

SD: standard deviation. CG: control group. EG: exposed group. OPMDG: oral potential malignant disorder group. OSCCG: oral squamous cell carcinoma group. NI: not informed.

Most studies analyzing exfoliated oral cells using the AgNOR technique refer to Ploton et al.^27^ for sample preparation and Crocker et al.^28^ for quantification (Table 5).

When considering several variables in AgNOR staining and interpretation, such as fixative solutions (propanol/carbowax, alcohol, isopropanol/methanol/ethylene glycol), silver staining times (20, 40, and 55 min), and the number of cells analyzed (30, 50, and 100), it was evident that rigorous training and team calibration significantly improved standardization. This is supported by the studies of da Silva et al.^22^ and Paiva et al.;^23^ compared with our results, where despite differences in technique and processing, the outcomes were remarkably similar. This similarity may be attributed to the involvement of the same research group and senior trainers, indicating potential subjectivity in the analysis. Therefore, automated analyses could help decrease this bias in human interpretation. Using computer-assisted AgNOR histopathological analysis, Nararyanan et al.^24^ demonstrated mean AgNOR/nucleus values of 2.32 in the control group, 5.30 in the oral leukoplakia group, and 8.69 in the OSCCG. Compared to our cytopathology results using similar methodologies, the control group samples obtained similar scores. However, OPMDG and OSCCG showed lower values in cytopathological analysis. Increased cell proliferative activity was evident in OPMD and malignant lesions in cytopathological or histopathological analyses compared with normal samples.

The results obtained in our study indicated a concordance rate exceeding 80% between human analysis and the system if the mean count of AgNORs in each group was considered. Our previous study^13^ using the same system observed that counting nuclei and AgNORs achieved precision and recall of 0.94 and 0.90 for the nucleus and 0.82 and 0.74 for AgNORs, respectively. Computerized tools can shorten the time of analysis compared to humans.^30^

This study has some limitations. First can be the technique employed; some authors affirm that LBC results in the formation of a thin layer with higher mean smear cellularity, ensuring uniform distribution, reducing cellular overlap, and providing clearer background due to decreased blood, saliva, microbial colonies, and debris compared with CC, which is important to differentiate NORs.^21,31^ Remmerbach et al. compared CC and LBC in oral mucosal brush biopsies with sensitivity of 96.3% and 97.5%, respectively, and specificity of 90.6% and 68.8%, respectively.^32^ Meanwhile, Alsarraf et al. showed an accuracy of 75% in analyzing LBC samples of non-dysplastic and dysplastic epithelial, OSCC, and oral lichen planus correlated with histopathological diagnosis.^33^ In another study with a highly representative sample, Kokubun et al. demonstrated that the sensitivity, specificity, positive predictive value, and negative predictive value of the cytological diagnosis of OSCC using LBC were 69%, 75%, 38%, and 92%, respectively.^34^ The results may be controversial, and a histopathological diagnosis should be made in patients with clinical suspicion, even if the cytological diagnosis is negative for malignancy.

Second, scanning slides can significantly reduce processing time because it eliminates the need to take isolated photographs of portions of the sample. Access to these resources is gradually spreading worldwide and may progressively increase and enhance research in this field. Conversely, the AgNOR technique is simpler and less expensive than the immunocytochemical techniques used to evaluate proliferation rates, such as P53 and PCNA. Other oral cytopathological analyses that can be used for cancer screening include assessing cellular maturation patterns and cytomorphometry with Papanicolaou staining, DNA-cytometric examination via the Feulgen reaction, and molecular analyses using mass spectrometry and DNA-image cytometry, which can detect significant alterations in cellular DNA content such as aneuploidy^35^. Our research group has continuously explored solutions that are applicable for large-scale use in countries with limited financial resources and the use of simple and less expensive techniques such as AgNOR and Pap staining to improve the collection, processing, and analysis of samples.

The developed automated model enabled the quantification of cells with one, two, three, four, and five or more NORs per nucleus, allowing faster analysis of proliferation rates than manual human analysis. The potential to obtain longitudinal results with an automated output is a noteworthy advantage. The CNN system can assess risk through a progressive increase in these counts over time or by evaluating the volumes of NORs.^36,37^

## Conclusion

This study proposes that AgNOR measurements can serve as markers for suspected epithelial cells in the oral mucosa. The suggested cut-off point of greater than 3.69 NORs per nucleus provides good accuracy in detecting suspicious malignant cells. Establishing cut-off points offers a numerical argument to alert patients exposed to OSCC risk factors and those with potentially malignant disorders. The CNN AgNOR System – Slide Examiner was able to differentiate the nuclei and satisfactorily count the number of NORs, demonstrating good reliability between the AgNORs assigned by the system and the human count. An automated approach may also be associated with other clinical parameters or longitudinal evaluations to achieve more accurate performance. After establishing this automated methodology, its potential application in clinical workflows, including primary care or oral cancer screening programs, expanded as the technique was refined using new resources over time.
